# New Calix[4]arene—Fluoresceine Conjugate by Click Approach—Synthesis and Preparation of Photocatalytically Active Solid Lipid Nanoparticles

**DOI:** 10.3390/molecules27082436

**Published:** 2022-04-09

**Authors:** Vladimir A. Burilov, Alina A. Artemenko, Ramilya I. Garipova, Rezeda R. Amirova, Aigul M. Fatykhova, Julia A. Borisova, Diana A. Mironova, Elza D. Sultanova, Vladimir G. Evtugyn, Svetlana E. Solovieva, Igor S. Antipin

**Affiliations:** 1 Organic and Medical Chemistry Department, Kazan Federal University, 18 Kremlevskaya St., 420008 Kazan, Russia; ultrav@bk.ru (V.A.B.); alinkamorning@gmail.com (A.A.A.); aukhadieva.ramilya@yandex.ru (R.I.G.); da.da.rezeda@mail.ru (R.R.A.); aigulfatykhova@gmail.com (A.M.F.); juuuliya99@mail.ru (J.A.B.); mir_din@mail.ru (D.A.M.); elsultanova123@gmail.com (E.D.S.); vevtugyn@gmail.com (V.G.E.); iantipin54@yandex.ru (I.S.A.); 2 Alexander E. Arbuzov Institute of Organic & Physical Chemistry, 8 Arbuzov Str., 420088 Kazan, Russia

**Keywords:** calixarene, fluorescein, luminescence, solid lipid nanoparticles, click chemistry, photocatalysis

## Abstract

New fluorescent systems for photocatalysis, sensors, labeling, etc., are in great demand. Amphiphilic ones are of special interest since they can form functional colloidal systems that can be used in aqueous solutions. A new macrocycle platform for click chemistry and its adduct with o-propargylfluoresceine was synthesized and characterized using modern physical techniques. Nanosized solid lipid nanoparticles (SLNs) from the calixarene—fluoresceine adduct were synthesized through the solvent injection technique and well-characterized in the solution and in solid state using light-scattering and microscopy methods. The maximum fluorescence intensity of the SLNs was found to be in the pH range from 7 to 10. The Förster resonance energy transfer (FRET) efficiency from SLNs to rhodamine 6g was found to be 97.8%. Finally, pure SLNs and the FRET system SLNs—Rh6G were tested in model photocatalytic ipso oxidative hydroxylation of phenylboronic acid under blue LED light. The SLNs—Rh6G system was found to be the best, giving an almost qualitative phenol yield, which was shown by HPLC-UV analysis.

## 1. Introduction

In the past few decades, the outstanding achievements of supramolecular chemistry—the chemistry beyond the molecule—have lead to a new tool for creating various functional devices that work according to the principles of non-covalent supramolecular interactions [1,2,3]. Such systems can be used for applications such as molecular machines, catalysis, smart materials, and various types of sensors. Of particular interest are supramolecular objects with fluorescent properties. These systems find application both as chemosensors [4,5,6], and as a means for the selective labeling of cells and other biological objects [7,8], and as unique catalytic systems for photocatalysis [9]. Such systems are somewhat more complex than conventional fluorescent molecules and, in addition to the fluorescent module, must have the property that ensures their supramolecular interactions with the object. For example, such molecules can be prone to supramolecular interactions of the guest–host type, which increase the recognition selectivity in the case of creating supramolecular chemosensors. To facilitate penetration into cells in the case of biolabeling, as well as to increase the number of binding centers in the case of creating biosensors operating in aqueous media, such fluorescent supramolecular objects should have an amphiphilic architecture. 

One of the most convenient platforms for obtaining amphiphilic structures are calixarenes [10,11,12,13]. The simplicity of their chemical modification, as well as the variety of stereoisomeric forms, make it possible to build completely diverse supramolecular systems on their framework. Fluorescent adducts of calixarenes are no exception, as discussed in detail in Kumar’s review [14]. A particularly convenient tool for their modification is popular click chemistry, the reaction of azide–alkyne cycloaddition [15]. We have previously demonstrated that both classical calixarenes and thiacalixarenes can be used to obtain amphiphilic structures with various polar functional groups using click chemistry [16,17,18,19].

Despite the abundance of publications related to fluorescent adducts of calixarenes, there are practically no publications related to the preparation of amphiphilic fluorescent adducts and colloidal supramolecular systems based on them. In this regard, the present work is devoted to the development of methods for the synthesis of adducts of classical calixarene with fluorescein using click chemistry, as well as the preparation of stable colloidal fluorescent systems based on them for photocatalytic applications.

## 2. Results and Discussion

**Scheme 1:**
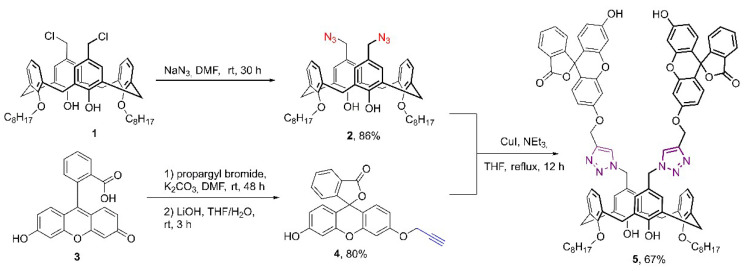
Synthesis of calix[4]arene (**5**), containing moiety of fluorescein in lactones form.

Recently [20], we modified Loy’s method of the chloromethylation of bis-alkyl calix[4]arene derivatives [21] to prepare bis-chloromethylated calixarene **1** with a high yield. This type of macrocycle contains a highly mobile benzyl chlorine atom; that is why it can be easily substituted with different nucleophiles. To prepare the CuAAC calixarene precursor, we used calixarene **1** in a reaction with excess NaN_3_ in DMF (Scheme 1). After 30 h of mixing, bis-azide **2** was isolated as a white solid with a high yield (86%). 

**Figure 1:**
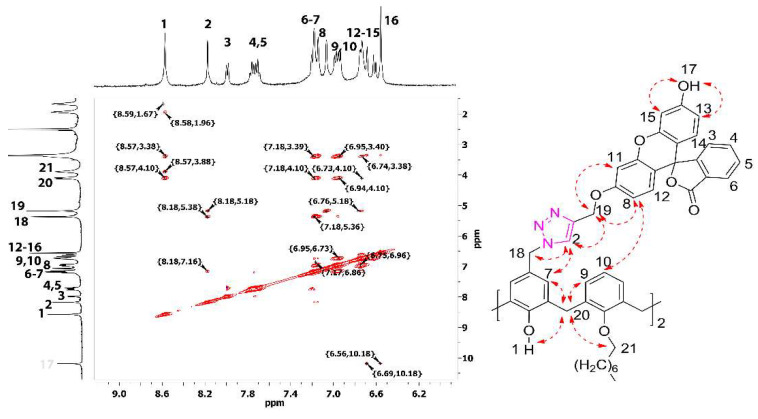
Fragment of the ^1^H–^1^H NOESY NMR spectra of **5** (DMSO–d_6,_ 25 °C).

The structure of bis-azide **2** was well-proven using NMR ^1^H, ^13^C, IR, and high-resolution ESI (HRESI) mass spectrometry. In the ^1^H NMR spectra, the most significant upfield change was undergone by the signal of CH_2_N_3_ protons (∆δ = 0.33 ppm) compared to starting calixarene **1** (Figure S1a). In the IR spectra of **2,** the presence of the azide groups was confirmed by specific valent asymmetric bond vibrations at 2103 cm^−1^ (Figure S1c). According to the HRESI MS data, there is the presence of a [M + Na]^+^ ion (m/z calculated for C_46_H_58_N_6_O_4_Na = 781,4417, found to be 781,4421), and the most intensive ion was [M+K]^+^ (calcd. for C_46_H_58_N_6_O_4_K = 797,4157, found to be 781,4156) (Figure S1d). The obtained macrocycle **2** was used in a CuAAC reaction with two equivalents of o-propargylfluorescein **4**, obtained using the literature method from fluorescein **3** by di-propargylation with subsequent ester hydrolysis with LiOH [22]. The calixarene–fluoresceine adduct **5** was isolated in a 67% yield after column chromatographic purification. The assignment of signals in the ^1^H NMR spectrum, as well as the confirmation of the stereoisomeric form, were carried out using the NMR ^1^H-^1^H 2D NOESY experiment. In the corresponding spectra, there are cross-peaks between protons of the phenolic hydroxyl group with protons of the hydroxymethyl group of octyoxy substituents in the lower rim (δ = 8.57 and 3.88 ppm, respectively) as well as the cross-peaks between calixarene aromatic protons of the neighboring benzene units (δ = 7.18 and 6.86 ppm, respectively), which clearly indicates the *cone* stereoisomeric form of the macrocycle. Other important cross-peaks are marked in Figure 1 and include the proximity of the triazole protons with the two methylene linkers between calixarene and fluoresceine moieties (δ = 8.18 with 5.18 and 5.38 ppm, respectively). Calixarene aromatic protons also have a cross-peak with the xantene part of fluoresceine (δ = 6.96 with 6.75 ppm). Additionally, the *cone* stereoisomeric form of the macrocycle **5** can be confirmed by the difference in the chemical shifts of the axial and equatorial methylene protons of the calixarene core (Gutche’s rule [23])—∆δ should be in the range of 0.7–1.0 ppm (herein ∆δ = 0.72 ppm).

In the IR spectra of the macrocycle **5** (Figure S2, c), there is intensive band at 1766 cm^−1^, which can be attributed to the stretching vibrations of the lactone carbonyl group [24] as well as the intensive band at 1612 cm^−1^, which can be attributed to the stretching vibrations of the xantene aromatic skeletal [25]. According to the HRESI MS [M+H]^+^ (calcd. for C_92_H_87_N_6_O_14_ = 1499.6275, found to be 1499.6280) and [M + Na]^+^ (calcd. for C_92_H_86_N_6_O_14_Na = 1521.6100, found to be 1521.6083), ions were found (Figure S2d).

The resulting macrocycle was used for the synthesis of solid lipid nanoparticles (SLNs) by the solvent injection technique [26]. For this, different amounts of a THF solution of **5** (from 50 to 500 μL) were injected into 5 mL of TRIS buffer (pH 7.4). According to the dynamic-light-scattering method (Table 1), the average hydrodynamic particle diameter is about 100 nm with the monomodal intensity distribution of particles. Zeta potential values indicate the formation of stable SLNs with the negative charge of the core. It is well-known that fluorescein dyes undergo protolytic transformations in the aqueous and surfactant solutions and have ring–chain tautomerism [27]. Taking into account the pK_a_ values of fluorescein ether derivatives (pK_a1_ = 4.73 ± 0.01) [28], at pH 7.4, the equilibrium was significantly shifted towards the monoanionic form; this is confirmed by the high negative surface potential of the SLNs.

**Figure 2:**
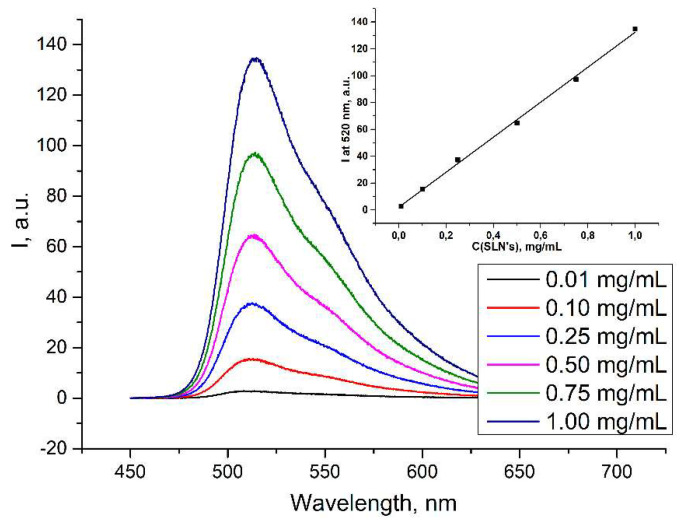
Emission spectra of SLNs (0.01–1 mg/mL) and emission intensity at 520 nm vs. SLN concentration (insert).

In the case of 3 mg/mL SLNs, the particle size and polydispersity index both increase. With an increase in the content of the **5** in SLNs, a linear increase in the intensity of the fluorescence emission in aqueous solutions was observed (Figure 2).

**Figure 3:**
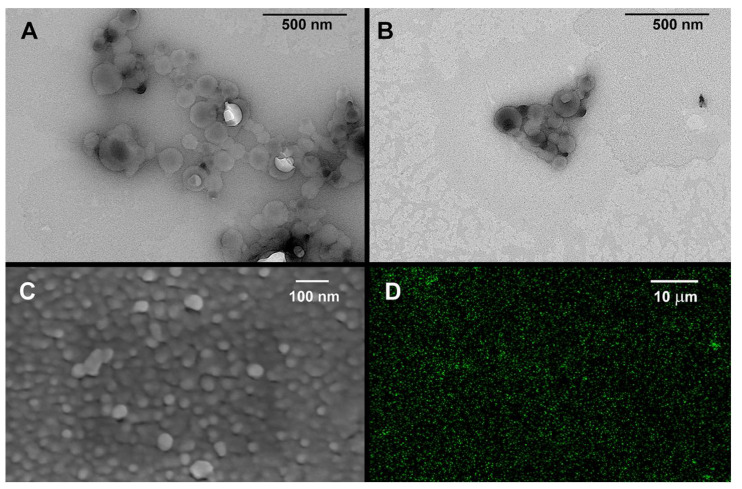
TEM (**A**, **B**), SEM (**C**) and confocal microscopy (**D**) images (λ_ex_ = 405 nm, 410–695 nm scan range) of SLNs from **5** (0.01 mg/mL).

According to the TEM and SEM microphotographs (Figure 3a–c) the mixture of SLNs was dominated by spherical particles with sizes of about 50–100 nm, but larger aggregates were also encountered.

Obtained SLNs were also studied using confocal laser microscopy (Figure 3d). The resulting micrograph shows quite evenly distributed nanoparticles with an intense green emission.

**Figure 4:**
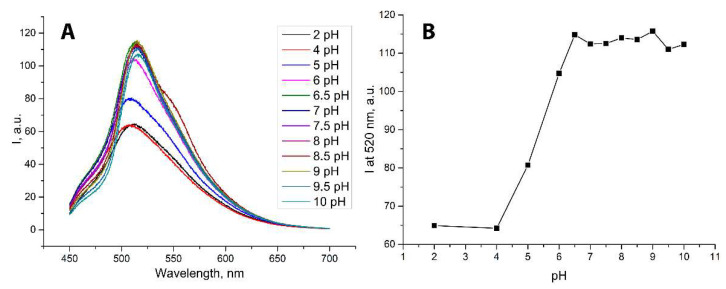
Emission spectra of SLNs (1 mg/mL) (**A**) and fluorescence intensity at 520 nm vs. pH (**B**).

Taking into account the protolytic transformation of the fluorescein dyes, we also examined the colloidal characteristics as well as the fluorescence intensity at different pH values. Fluorescence intensity has a clear pH dependence (Figure 4). For example, when passing from the acidic pH region to pH 7, a twofold increase in the particle emission intensity was observed.

The size and zeta potential are expected to change with the pH of the medium (Table 2). Thus, at pH 2, colloid-stable particles with a positive zeta potential are formed, which can be achieved by protonation of xanthene fragments (the conjugated acid of methyl-substituted fluorescein’s pK is 1.94 ± 0.01 logarithmic units [28]). Protonation of triazole fragments at pH 2 seems unlikely since the pK of a conjugated acid of 1,4 disubstituted 1,2,3, triazole is 0.05 ± 0.01 logarithmic units [29].

A sufficiently high emission of the obtained lipid particles, as well as the presence of a molecular macrocyclic cavity capable of interaction with guests, establishes the prerequisites for using them as a donor for energy transfer via Förster resonance energy transfer (FRET) to another dye [30]. The key requirement for an acceptor guest dye is that its absorption spectrum overlaps with the emission spectrum of the donor. Such systems are of great demand for the creation of highly sensitive chemosensors operating via changing the distance between the donor–acceptor pair [31], and also have prospects for use in photocatalysis [32]. Rhodamine 6g (Rh6G) was selected as a FRET acceptor for the SLNs since fluorescein—Rh6G is a well-known FRET pair with a FRET efficiency over 70% [33].

**Figure 5:**
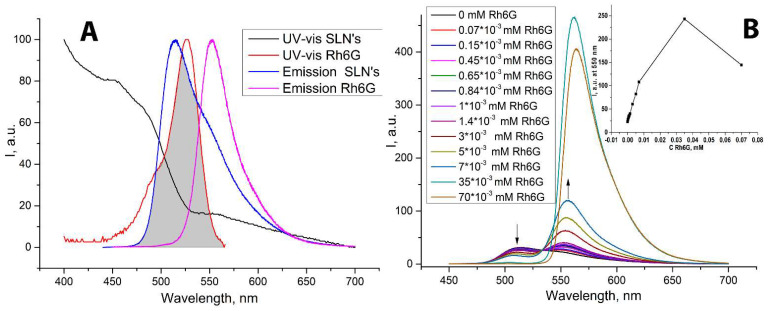
Normalized absorption and emission spectra of SLNs and Rh6G (**A**), emission spectra of SLNs (1 mg/mL) in the presence of various Rh6G concentrations (0.07 µM–0.07mM) (**B**). All emission spectra were registered using λ _ex_ = 430 nm.

According to the data obtained, the emission spectrum of the obtained nanoparticles has a significant overlap with the absorption spectrum of Rh6G, which is the prerequisite for the effective FRET in this system (Figure 5A). To study FRET, a steady-state fluorescent experiment was carried out in the presence of different amounts of an acceptor—Rh6G (Figure 5B). The FRET efficiency, E, was calculated using Formula (1):

E = 1 − I_DA_/I_D_ (1)

where I_DA_ and I_D_ are the donor (SLNs) fluorescence intensities (at 520 nm) in the presence and absence of Rh6G, respectively.

**Figure 6:**
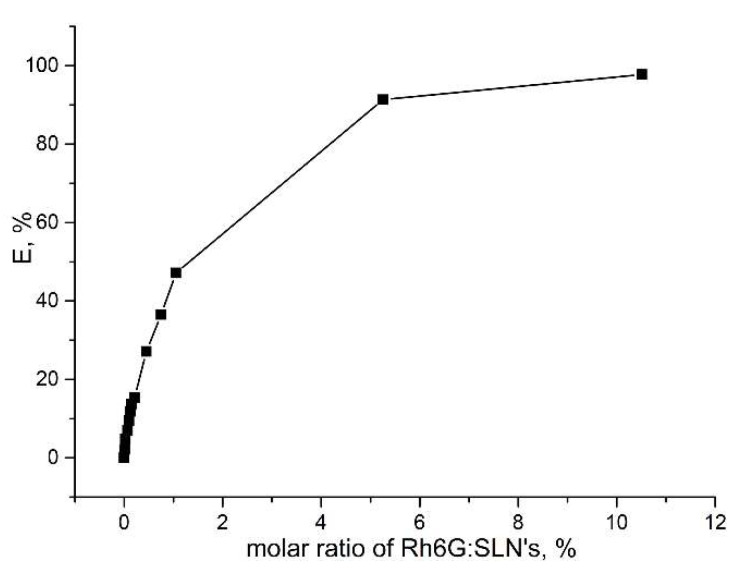
FRET efficiency E vs. molar ratio of Rh6G:SLNs, Rh6G 0.07 µM—0.07mM, SLNs 0.67 mM.

According to the calculated data (Figure 6), the maximum energy transfer of 97.8% is observed at a 0,1 excess of Rh6G vs. SLNs. It is important to note that, however, at 0.035 mM of Rh6G, the intensity of Rh6G emission increased nonlinearly (Figure 5, B, insert), and a slight bathochromic shift of the emission maximum was also observed (from 555 nm to 565 nm). An additional increase in the intensity of Rh6G is due to the fact that in addition to FRET excitation at these concentrations, direct excitation of Rh6G at 430 nm occurs (Figure S3). Such a bathochromic shift may indicate intense adsorption of the dye by the hydrophobic part of the SLNs, which is accompanied by observed solvatochromic effect. However, a further increase in the content of Rh6G to 0.07 mM induces a certain decrease in the emission intensity, which may be due to the effect of concentration quenching of the dye due to an increase in its local content on SLNs’ hydrophobic region, which was not observed in the case of the emission of free Rh6G at the same concentrations (Figure S3, insert). According to the data of one-dimensional and two-dimensional NMR spectroscopy, macrocycle **5** itself does not form a host–guest inclusion complex with Rh6G; there were no NMR shifts of the macrocycle—dye solutions in 1:1 stoichiometry and a lack of the NOESY cross-peaks between them (Figure S4). However, according to the literature data, the Förster distance for the fluorescein–Rh6G pair is about 5 nm [34,35]. Such a distance can be seen in the case when the dye is adsorbed on the surface of the SLNs, not necessarily forming an inclusion complex of the guest–host type. Taking into account the observed bathochromic shift in the Rh6G spectrum in the presence of SLNs, it is most likely that Rh6G is adsorbed in the hydrophobic zone of lipid particles. To evaluate adsorption efficiency, an Rh6G sorption experiment was carried out. To do this, 3 mL of a solution containing 1 mg/mL of SLNs was placed in a dialysis bag with 1 kDa pores and subjected to dialysis against 50 mL of distill water containing 0.007 mM Rh6G. After one hour, 52 % of the dye was adsorbed by SLNs, remaining unchanged over the next 24 h (Figure S5), thus confirming that the SLNs effectively adsorb and retain Rh6G dye.

**Scheme 2:**
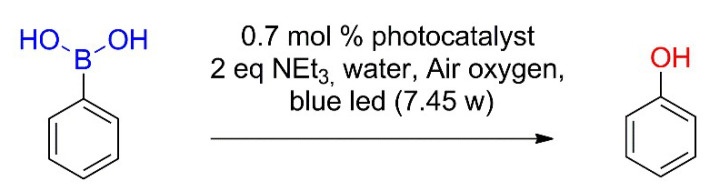
Photoredox hydroxylation of phenylboronic acid.

Finally, the SLNs and FRET system SLNs—Rh6G were tested in model photocatalytic ipso oxidative hydroxylation of phenylboronic acid (Scheme 2).

This photoredox reaction was first evaluated by Zou at al in 2012 using [Ru(bpy)_3_]Cl_2_ as a single-electron transfer (SET) agent and sacrificial triethylamine as an electron donor [36]. Later, metal-free oxidative hydroxylation of phenylboronic acids was presented using various SET agents, such as methylene blue [37], fullerene [38], etc. To the best of our knowledge, fluorescein and its derivatives have not been used in the oxidative hydroxylation of arylboronic acids despite the fact that fluorescein itself has been successfully used as a photosensitizer in another photoredox reaction [39].

The reaction was carried out in water for 48 h in the presence of atmospheric oxygen under blue LED light (Figure S6). The phenol yield was quantitated using the HPLC–UV absolute calibration method (Figure S7). The advantage of HPLC–UV in monitoring this reaction can also be attributed to the fact that it is possible to monitor both phenol and the starting phenylboronic acid, which distinguishes the method from GCMS (Figure S8). Free fluorescein as well as Rh6G exhibited low catalytic activity under these conditions (Table 3). Nevertheless, SLNs demonstrated much higher phenol production. The best catalytic activity was demonstrated by the FRET system based on SLNs with Rh6G—in this case, a practically quantitative conversion of phenylboronic acid was achieved (Table 3, Figure S9), which is an outstanding result, given that the reaction was carried out in the presence of air oxygen unlike balloon oxygen in other publications. Reactions without a catalyst as well as in the absence of irradiation resulted in only trace amounts of phenol.

**Scheme 3:**
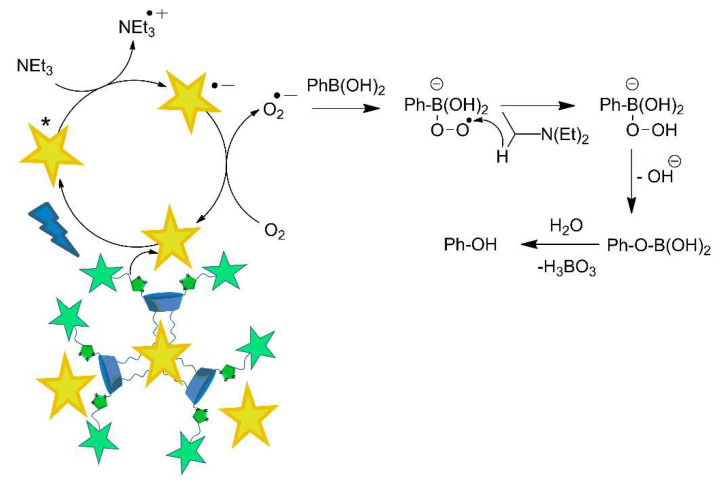
Proposed mechanism for the oxidative hydroxylation of phenylboronic acid using SLNs–Rh6G.

To understand the role of the macrocycle in increasing the activity of a catalyst, it is necessary to look at the reaction mechanism (Scheme 3). It is known [36,37,38] that the key step of the mechanism consists of the generation of superoxide radicals. Thus, in the first stage, SLN particles transfer energy to Rh6G through FRET. Rh6G goes into an excited state, and, through interacting with sacrificial triethylamine, turns into an anion radical. Air oxygen, reacting with the radical anion, turns into superoxide ion, which then reacts with phenylboronic acid. It is obvious that the SLNs catalyze the process even without Rh6G; therefore, the excitation and participation of fluorescein substituents in the process are also possible. Taking into account that calixarene-containing systems are able to stabilize reactive superoxide radical [40,41], observed acceleration of the reaction in the case of SLN-based photocatalysis may be the result of stabilization of the superoxide species by the macrocycle, as well as the operation of SLNs as hydrophobic nanoreactors.

## 3. Materials and Methods

Chemicals were purchased from commercial suppliers and used as received. Solvents were purified according to standard procedures. Substance purity and the process of reaction were monitored by TLC on Merck UV 254 plates and visualized by exposure to UV with a VL-6.LC lamp (Vilber, Marne-la-Vallée, France).

^1^H and ^13^C NMR spectra as well as 2D ^1^H-^1^H NOESY were recorded on Bruker Avance 400 Nanobay (Bruker Corporation, Billerica, MA, USA) with signals from residual protons of DMSO-d_6_ or CDCl_3_ as internal standard.

The melting points were measured using the OIptimelt MPA100 melting point apparatus (Stanford Research Systems, Sunnyvale, CA, USA).

IR spectra in KBr pellets were recorded on a Bruker Vector-22 spectrometer (Bruker Corporation, Billerica, MA, USA).

High-resolution mass spectra with electrospray ionization (HRESI MS) were obtained on an Agilent iFunnel 6550 Q-TOF LC/MS (Agilent Technologies, Santa Clara, CA, USA) under the following conditions: carrier gas—nitrogen, temperature 300 °C, carrier flow rate 12 L•min^−1^, nebulizer pressure 275 kPa, funnel voltage 3500 V, capillary voltage 500 V, total ion current recording mode, 100–3000 *m*/*z* mass range, and scanning speed 7 spectra•s^−1^.

HPLC—UV determination of phenol was performed on VWR Hitachi Chromaster HPLC system (Hitachi High-Tech Corporation, Tokyo, Japan), equipped with L-2130 pump, L-2400 UV detector, and a 5310-column oven with Macherey-Nagel EC 250/4.6 NUCLEODUR C18 column (Macherey-Nagel GmbH, Duren, Germany) using 1.5 mL/min CH_3_CN-H_2_O (80:20) eluent.

TEM was performed on Hitachi HT7700 ExaLens (Hitachi High-Tech Corporation, Tokyo, Japan) in Interdisciplinary Center for Analytical Microscopy of Kazan Federal University. The images were acquired at an accelerating voltage of 100 kV. Samples were ultrasonicated in water for 10 min, dispersed on 200 mesh copper grids with continuous formvar support films, and then dried for 3 h.

SEM was performed using Merlin (Carl Zeiss, Jena, Germany). To minimize the effect on the object, the morphology was observed in the secondary electron (SE) mode with an accelerating voltage of primary electrons of 5 kV and a probe current of 300 pA.

Confocal laser microscopy images were obtained by CLMS on an inverted Carl Zeiss LSM 780 confocal laser scanning microscope (Carl Zeiss, Jena, Germany).

DLS and ELS experiments were carried out on Zetasizer Nano ZS instrument (Malvern Panalytical, Worcestershire, UK) with 4 mW 633 nm He–Ne laser light source and the light-scattering angle of 173°. The data were treated with DTS software (Dispersion Technology Software 5.00). The solutions were filtered through 0.8 μM filter before the measurements to remove dust. The experiments were carried out in the disposable plastic cells DTS 0012 (size) or in the disposable folded capillary cells DTS 1070 (zeta potential) (Sigma–Aldrich, Burlington, MA, USA) at 298 K with at least three experiments for each system.

UV-visible spectra were recorded using Shimadzu UV-2600 spectrophotometer equipped with Shimadzu TCC-100 thermostat (Shimadzu Corporation, Kyoto, Japan).

Fluorescence spectra were performed in 10.0 mm quartz cuvettes and recorded on a Fluorolog FL-221 spectrofluorimeter (HORIBA Jobin Yvon, Kyoto, Japan) using excitation wavelength 430 nm with 1 nm slit. All studies were conducted at 298 K.

11, 23-*Bis*(chloromethyl)-25,27-dihydroxy-26,28-dioctyloxycalix[4]arene (**1**) and o-propargylfluorescein (**4**) were synthesized according to the literature procedures [20,22].

Synthesis of 11,23-Bis(azidomethyl)-25,27-dihydroxy-26,28-dioctyloxycalix[4]arene (**2**): 11,23-*Bis*(chloromethyl)-25,27-dihydroxy-26,28-dioctyloxycalix[4]arene (**1**) (1.04 g, 1.4 mmol) and NaN_3_ (0.36 g, 5.7 mmol) were dissolved in 20 mL of dry DMF. Reaction mixture was stirred at rt for 30 h. The completion of the reaction was determined by TLC (hexane:EtOAc = 2:1). Then, 30 mL of CH_2_Cl_2_ was added to the reaction mixture, and organic layer was washed with 1M HCl (20 mL). Then, organic layer was washed with water (3 * 20 mL) and dried over anhydrous Na_2_SO_4_. Solvents were removed by rotary evaporator to give **2** as a white solid. Yield 0.91g (86%). TLC R_f_ = 0.5 (hexane:EtOAc, 2:1); mp 118 °C. ^1^H NMR (400 MHz, CDCl_3_, 25 °C): δ, ppm: 0.90 (t, *J* = 6.5 Hz, 6H, -CH_2_-CH_3_), 1.28–1.49(m, 16H, -CH_2_-),1.79–1.61 (m, 4 H, -CH_2_-CH_3_), 1.91–2.14 (m, 4H, -OCH_2_-CH_2_-), 3.39 (d, *J* = 13.0 Hz, 4H, CH_2_), 4.00 (t, *J* = 6.6 Hz, 4H, O-CH_2_), 4.18 (s, 4 H, CH_2_-N_3_), 4.31 (d, *J* = 12.9 Hz, 4H, CH_2_), 6.78 (t, *J* = 7.5 Hz, 2H), 6.94 (d, *J* = 7.5 Hz, 4H, Ar), 7.01 (s, 4H, Ar), 8.44 (s, 2H, OH).

^13^C NMR (100.6 MHz, CDCl_3_, 25 ^o^C): δ, ppm: 153.71, 152.10, 133.27, 129.17, 128.76, 128.55, 125.60, 125.47, 54.81, 32.10, 31.56, 30.16, 29.65, 29.49, 26.13, 22.84, 14.28. IR (KBr) ν _max_ cm^−1^: 3331 (br, OH), 2926, 2855, 2103 (N_3_), 1483, 1457, 1248, 1168, 1077, 761. HRESI MS (*m*/*z*) [M + Na]^+^: calcd. for C_46_H_58_N_6_O_4_Na = 781,4417, found to be 781,4421; [M + K]^+^: calcd. for C_46_H_58_N_6_O_4_K = 797,4157, found to be 781,4156.

Synthesis of 11,23-Bis-((4-(((3′-hydroxy-3-oxo-3H-spiro[isobenzofuran-1,9′-xanthen]-6′-yl)oxy)methyl)-1H-1,2,3-triazol-1-yl)methyl)-25,27-dihydroxy-26,28-dioctyloxycalix[4]arene (**5**): the solution of 0.1 g (0.135 mmol) of 11,23-*bis*(azidomethyl)-25,27-dihydroxy-26,28-dioctyloxycalix[4]arene **2** in THF (5 mL) was added to NEt_3_ (0.5 mL) followed by 0.1 g (0.27 mmol) of o-propargylfluorescein (**4**) and 2.6 mg (0.0135 mmol) of CuI. The reaction mixture was refluxed for 12 h with TLC control (hexane:EtOAc = 2:1). After completion of the reaction, solvent was removed using rotary evaporator and the obtained crude product was purified by SiO_2_ column chromatography (CHCl_3_ with 5% MeOH) to give calixarene **5** as pale yellow solid. Yield 0.135 g (67%). TLC R_f_ = 0.2 (hexane:EtOAc, 2:1); mp 192 °C (decomp.). ^1^H NMR (400 MHz, DMSO-d_6_, 25 °C): δ, ppm: 0.68–0.91(m, 6 H, -CH_2_-CH_3_), 1.09–1.47 (m., 16H, -CH_2_-), 1.57–1.74 (m, 4H, -CH_2_-CH_3_), 1.83–2.02 (m, 4H, -OCH_2_-CH_2_-), 3.40 (d, *J* = 11.7 Hz, 4H, Ar-CH_2_-Ar), 3.74–3.98 (m, 4H, O-CH_2_-CH_2_-), 4.10 (d, *J* = 12.1 Hz, 4H, Ar-CH_2_-Ar), 5.18 (s, 4H, -CH_2_-O-Flu), 5.37 (s, 4H, -CH_2_-Trz), 6.56 (s, 2H, Ar_Flu_), 6.59–6.78 (m, 8H, Ar_Flu_), 6.88–7.02 (m, 6H, Ar), 7.06 (brs, 2H, Ar_Flu_), 7.11–7.24 (m, 6H, Ar + Ar_Flu_), 7.67–7.79 (m, 4H, Ar_Flu_), 7.99 (d, *J* = 7.7 Hz, 2H, Ar_Flu_), 8.18 (s, 2H, Trz), 8.57 (s, 2H, OH_cal_), 10.19 (s, 2H, OH_Flu_); ^13^C NMR (100.6 MHz, DMSO-d_6_, 25 °C): δ, ppm 168.60, 159.70, 159.54, 152.73, 152.39, 151.73, 142.43, 135.60, 133.39, 130.12, 129.05, 128.91, 128.64, 127.99, 126.14, 126.02, 125.34, 124.64, 124.42, 123.94, 112.77, 112.44, 111.24, 109.37, 102.19, 101.57, 76.44, 61.39, 52.62, 31.36, 30.33, 29.54, 28.89, 28.76, 25.38, 22.10, 13.91. IR (KBr) ν _max_ cm^−1^: 3321 (br, OH), 2924, 2852, 1766, 1612, 1461, 1249, 1179, 1108, 1012, 761. HRESI MS (*m*/*z*) [M+H]^+^: calcd. for C_92_H_87_N_6_O_14_ = 1499.6275, found to be 1499.6280; [M+Na]^+^: calcd. for C_92_H_86_N_6_O_14_Na = 1521.6100, found to be 1499.6083.

### 3.1. Preparation of SLNs

Different amounts (from 50 to 500 μL) of a THF stock solution of **5** (C = 6.0 mM) were injected into 5 mL of 10 mM TRIS buffer (pH = 7.4) or universal Britton–Robinson buffer under vigorous stirring. After stirring for 15 min, THF was evaporated in vacuo.

### 3.2. Photocatalytic Oxidation of Phenylboronic Acid

In a glass vial, 0.1 mmol of phenylboronic acid, 0.2 mmol of triethylamine and 1 mL of buffer solution containing photocatalyst (0.7 µmol of Fluorescein, Rh6G and SLNs or 0.7 µmol SLNs + 0.035 µmol Rh6G) were mixed together. The mixture was kept under blue LED irradiation (7.45 W) bubbling with air for 48 h.

## 4. Conclusions

A new macrocyclic precursor for click chemistry—calixarene bis-azide and its conjugate with o-propargylfluorescein—were synthesized for the first time. The obtained conjugate was used for the preparation of fluorescent solid lipid nanoparticles, which were obtained by the solvent injection technique and well-characterized by several microscopy methods. Obtained solid lipid nanoparticles were found to be good donors in the FRET pair with Rh6G with an efficiency of up to 97.8%. Pure SLNs and the SLNs—Rh6G system were tested in model photocatalytic ipso-oxidative hydroxylation of phenylboronic acid. Compared to all tested systems, including fluoresceine, Rh6G, and its FRET pair, the SLNs—Rh6G system was found to be the best, giving an almost qualitative phenol yield. The role of the calixarene core in the stabilization of reactive superoxide radical, which is generated during the reaction, is the most likely reason for the observed increase in the catalytic activity.

## Figures and Tables

**Table 1:** CAC values and DLS, and ELS data of SLNs at different concentrations ^1^.

C of SLNs, mg/mL	d, nm	PDI	ζ, mV
0.01	152 ± 19	0.339 ± 0.073	−21 ± 4
0.1	98 ± 1	0.194 ± 0.008	
0.25	97 ± 1	0.146 ± 0.017	
0.5	100 ± 1	0.145 ± 0.013	−30 ± 1
0.7	109 ± 1	0.137 ± 0.009	
1.0	116 ± 1	0.134 ± 0.012	−31 ± 1
3.0	541 ± 22	0.455 ± 0.043	−25 ± 3

^1^ C (Pyrene) in CAC experiment = 1 µM, C (1 = 2) = 0.1 mM, 10 mM TRIS.

**Table 2:** CAC values, and DLS and ELS data of SLNs at different pHs ^1^.

pH	d, nm	PDI	ζ, mV
2	92 ± 1	0.115 ± 0.009	+30 ± 8
4	458 ± 147	0.499 ± 0.134	+8 ± 1
5	3357 ± 3647	0.927 ± 0.110	−18 ± 3
6	749 ± 440	0.673 ± 0.257	−16 ± 1
6.5	144 ± 1	0.095 ± 0.019	−14 ± 1
7.1	226 ± 2	0.253 ± 0.005	−24 ± 1
7.5	100 ± 1	0.133 ± 0.010	−27 ± 1
8	101 ± 1	0.162 ± 0.006	−27 ± 1
8.6	78 ± 1	0.192 ± 0.003	−27 ± 1
9	77 ± 1	0.241 ± 0.011	−29 ± 1
9.5	57 ± 1	0.328 ± 0.011	−29 ± 1
10	64 ± 1	0.387 ± 0.017	−20 ± 2

^1^ C (SLNs) = 0.07 µM, 10 mM universal Britton–Robinson buffer.

**Table 3:** HPLS–UV yield of phenol from photoredox hydroxylation of phenylboronic acid using different catalytic systems ^1^.

System	Phenol Yield, % ^2^
Fluorescein	6
Rh6G	3
Fluorescein + Rh6G	22
SLNs	64
**SLNs+Rh6G**	**96**
SLNs+Rh6G no light	1
no photocatalyst	1

^1^ 0.1 mmol PhB(OH)_2,_ 0.2 mmol NEt_3_, 0.7 µmol photocatalyst (Fluorescein, Rh6G and SLNs) or 0.7 µmol SLNs + 0.035 µmol Rh6G or 0.035 µmol Fluorescein + 0.035 µmol Rh6G, blue LED 7.45 W. ^2^ Phenol yield was quantitated using HPLC–UV.

## Data Availability

Samples of the compounds **2**, **5** are available from the authors.

## Supplementary Materials

The following supporting information can be downloaded at: https://www.mdpi.com/article/10.3390/molecules27082436/s1. Figure S1: NMR ^1^H (a), ^13^C (b), FT IR (c), and HRESI MS (d) spectra of 11,23-Bis(azidomethyl)-25,27-dihydroxy-26,28-dioctyloxycalix[4]arene (**2**); Figure S2: NMR ^1^H (a), ^13^C (b), FT IR (c), and HRESI MS (d) spectra of 11,23-Bis-((4-(((3’-hydroxy-3-oxo-3H-spiro[isobenzofuran-1,9’-xanthen]-6’-yl)oxy)methyl)-1H-1,2,3-triazol-1-yl)methyl)-25,27-dihydroxy-26,28-dioctyloxycalix[4]arene (**5**); Figure S3: Emission spectra of Rh6G (0.07 µM–0.07 mM); Figure S4: NMR ^1^H spectra of 11,23-Bis-((4-(((3’-hydroxy-3-oxo-3H-spiro[isobenzofuran-1,9’-xanthen]-6’-yl)oxy)methyl)-1H-1,2,3-triazol-1-yl)methyl)-25,27-dihydroxy-26,28-dioctyloxycalix[4]arene (**5**), and its mixture with Rh6G (a); 2D NMR ^1^H-^1^H NOESY spectra of **5**- Rh6G (b); Figure S5: UV-vis spectra of aqueous solution of Rh6G before sorption and after 1 h of 24 h of sorption by SLNs and photography of the sorption experiment setup (insert); Figure S6: Photoredox blue LED reactor setup; Figure S7: HPLC–UV calibration curve; Figure S8: Chromatograms of pure phenol (A) and reaction mixture of phenol and phenylboronic acid; Figure S9: HPLS–UV yield of phenol from photoredox hydroxylation of phenylboronic acid using SLNs+Rh6G catalytic system vs. time of irradiation.
