# Supplementary material for: New Calix[4]arene—Fluoresceine Conjugate by Click Approach—Synthesis and Preparation of Photocatalytically Active Solid Lipid Nanoparticles
Source: Molecules. 2022 Apr 9;27(8):2436. doi: 10.3390/molecules27082436 (PMC9028507; doi:10.3390/molecules27082436)
Supplement: Supplementary file 1 [file molecules-27-02436-s001.zip › molecules-1660350-supplementary.pdf]

New calix[4]arene – fluoresceine conjugate by click approach – synthesis and preparation of photocatalytically active solid lipid nanoparticles

Vladimir Burilov[1], Alina Artemenko[1], Ramilya Garipova[1], Rezeda Amirova[1], Aigul Fatykhova[1], Julia Borisova[1], Diana Mironova[1], Elsa Sultanova[1], Evtugyn Vladimir[1], Svetlana Solovieva[2*] and Igor Antipin[1]

1. Kazan Federal University, 18 Kremlevskaya st. Kazan, 420008, Russian Federation.
2. A.E.Arbuzov Institute of Organic & Physical Chemistry, 8 Arbuzov str., Kazan, 420088, Russian Federation.

* Correspondence: evgersol@yandex.ru, Tel.: (+7-843-2337344)

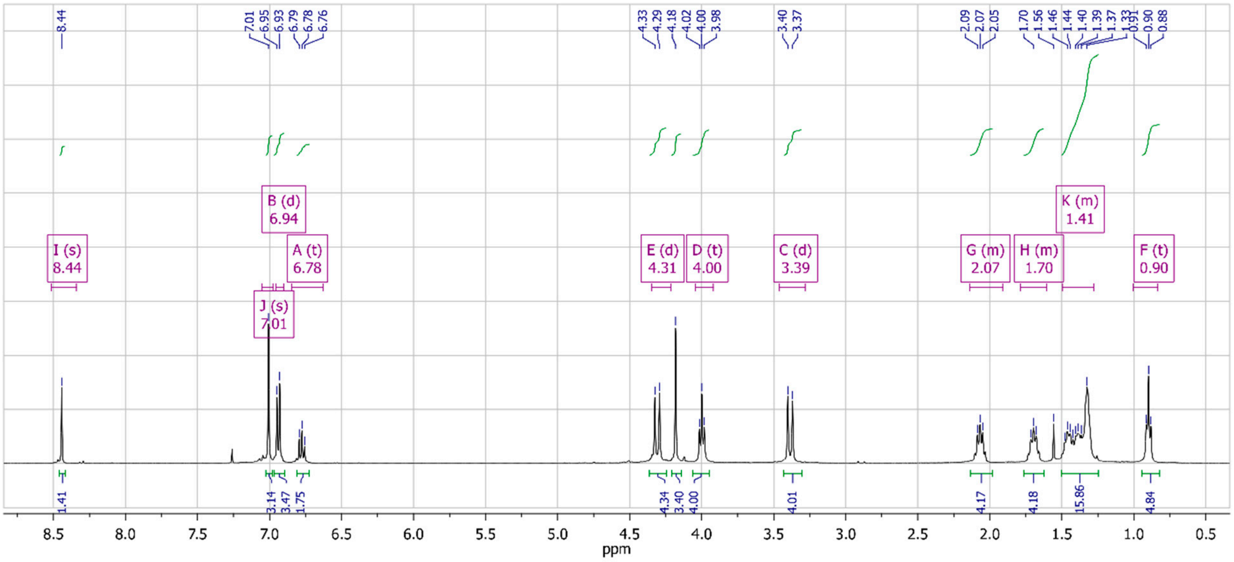

(**a**)

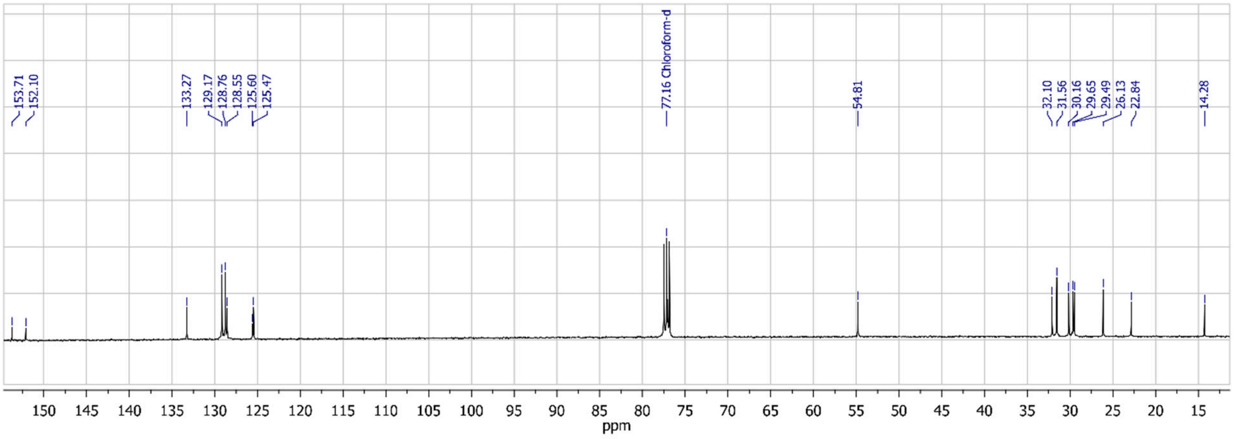

(**b**)

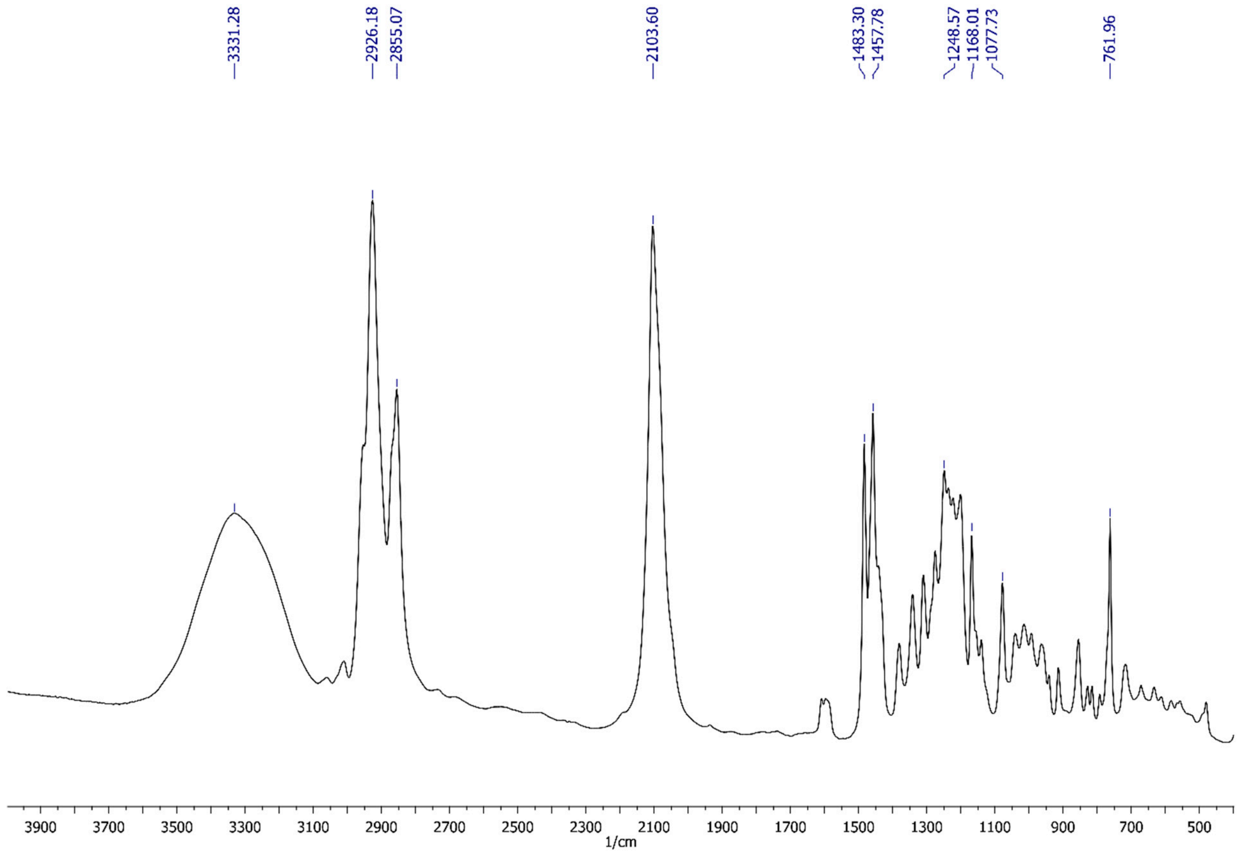

(**c**)

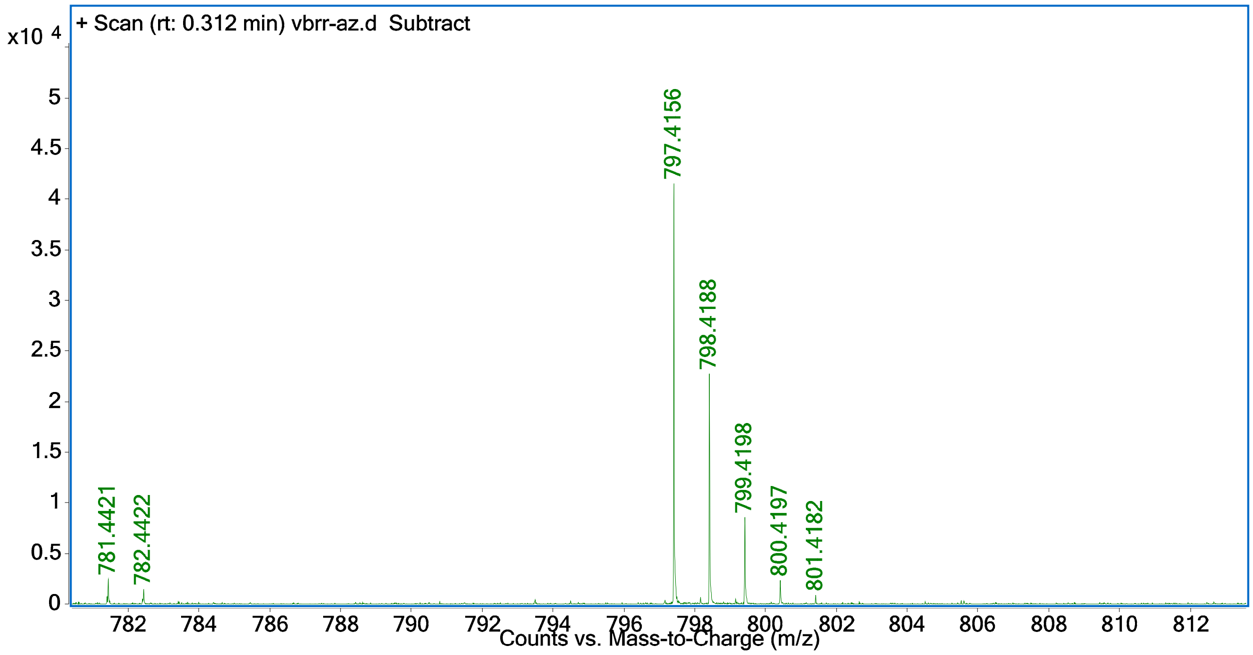

(**d**)

Figure S1. NMR ^{1}H (a), ^{13}C (b), FT IR (c) and HRESI MS (d) spectra of 11,23-Bis(azidomethyl)-25,27-dihydroxy-26,28-dioctyloxycalix[4]arene (**2**).

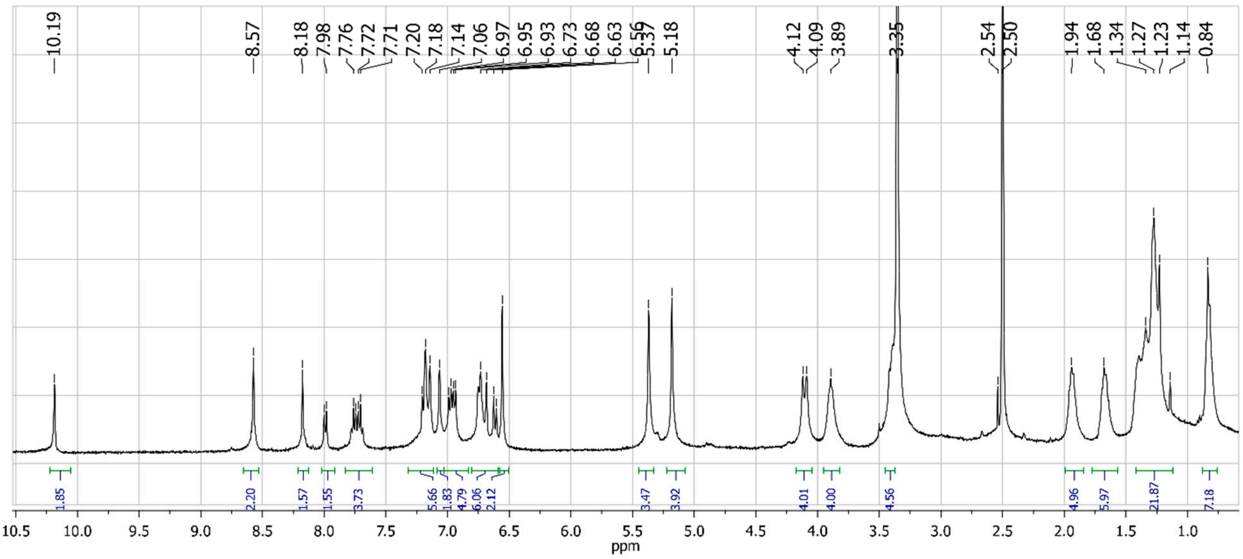
10.19
8.57
8.18
7.98
7.76
7.72
7.71
7.20
7.18
7.14
7.06
6.97
6.95
6.93
6.73
6.68
6.63
6.56
5.37
5.18
4.12
4.09
3.89
3.35
2.54
2.50
1.94
1.68
1.34
1.27
1.23
1.14
0.84
1.85
2.20
1.57
1.55
3.73
5.66
1.83
4.79
6.06
2.12
3.47
3.92
4.01
4.00
4.56
4.96
5.97
21.87
7.18
ppm

(**a**)

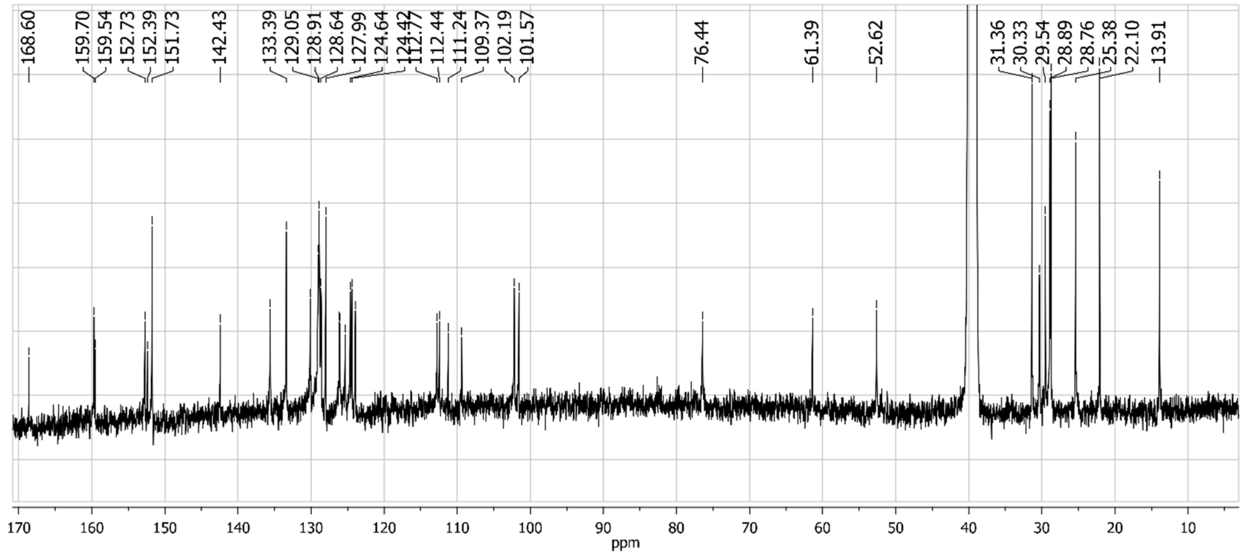
168.60
159.70
159.54
152.73
152.39
151.73
142.43
133.39
129.05
128.91
128.64
127.99
124.64
124.42
112.77
112.44
111.24
109.37
102.19
101.57
76.44
61.39
52.62
31.36
30.33
29.54
28.89
28.76
25.38
22.10
13.91
ppm

(**b**)

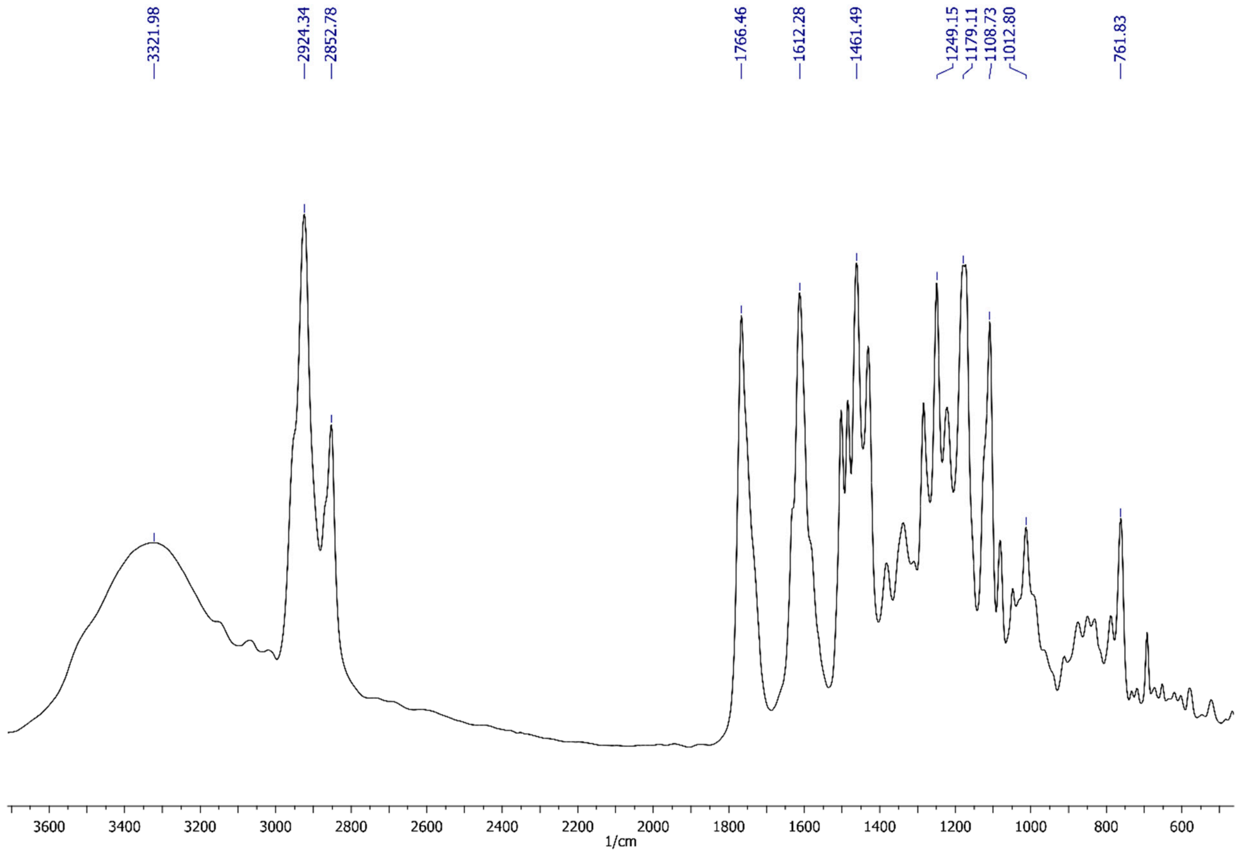

(c)

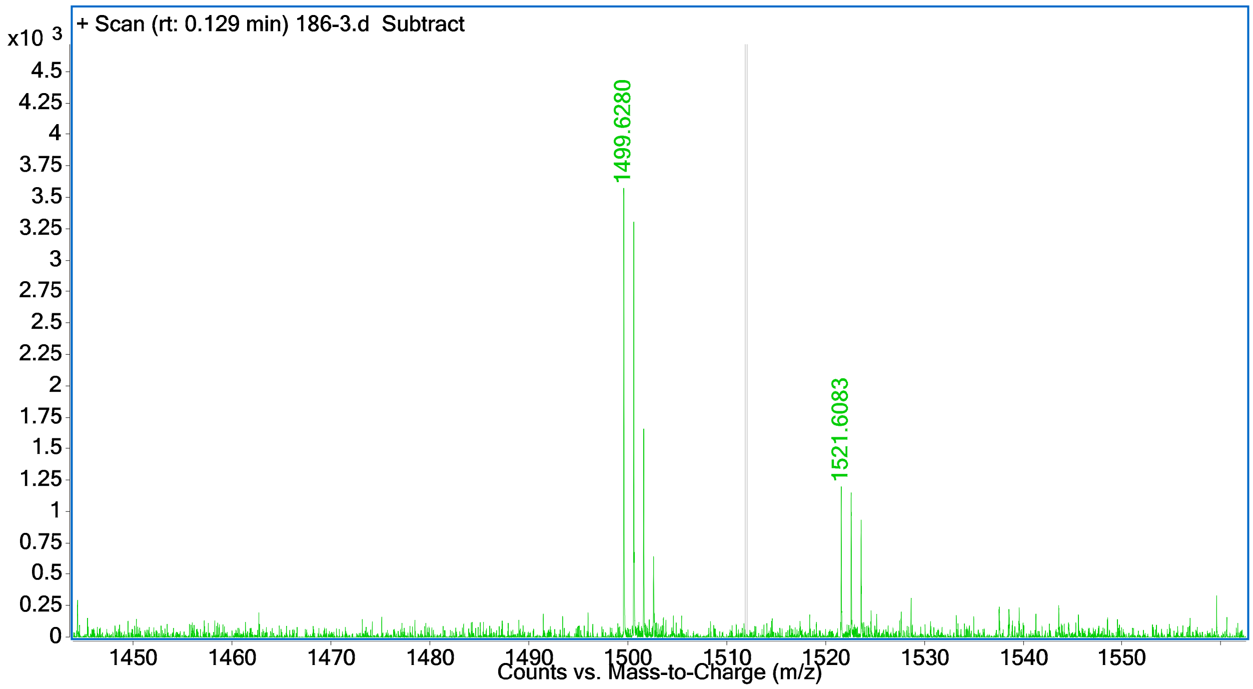

(d)

Figure S2. NMR ^{1}H (a), ^{13}C (b), FT IR (c) and HRESI MS (d) spectra of 11,23-Bis-((4-(((3'-hydroxy-3-oxo-3H-spiro[isobenzofuran-1,9'-xanthen]-6'-yl)oxy)methyl)-1H-1,2,3-triazol-1-yl)methyl)-25,27-dihydroxy-26,28-dioctyloxycalix[4]arene (**5**).

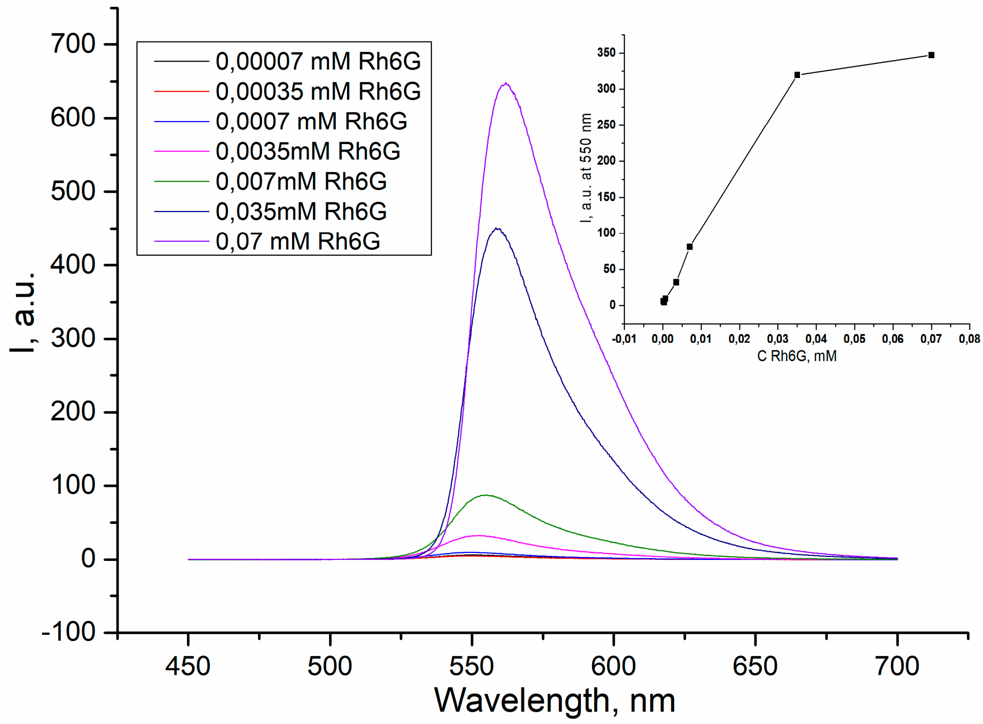

Figure S3. Emission spectra of Rh6G (0.07 μM - 0.07mM), λ ex = 430 nm.

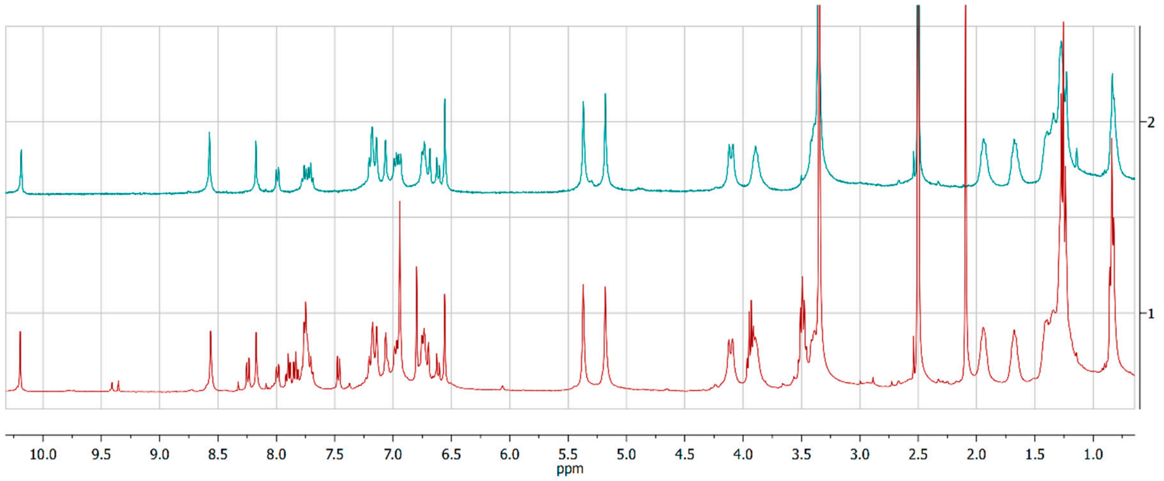

(a)

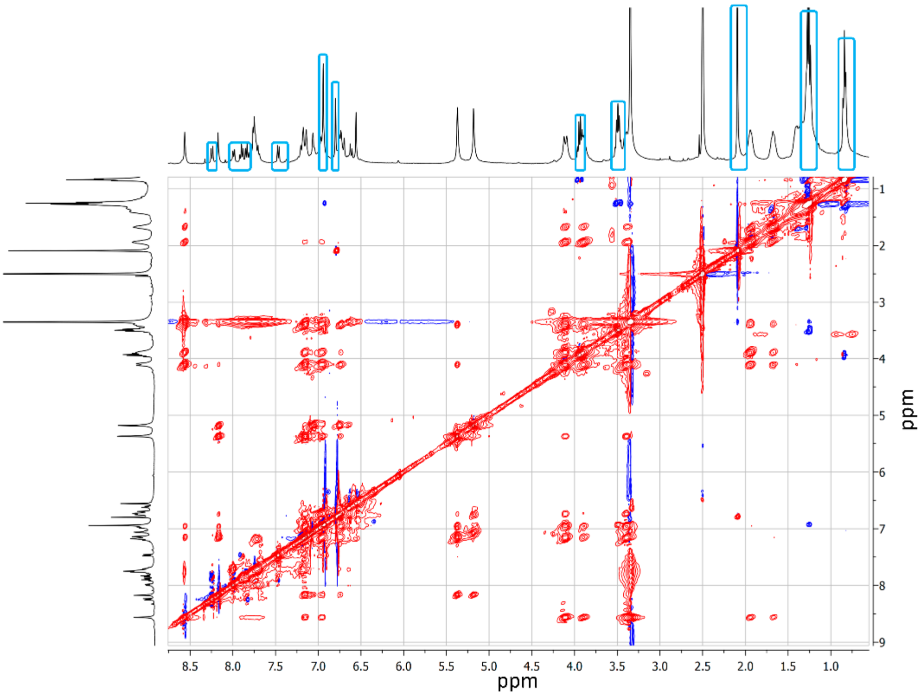

(b)

Figure S4. NMR ^{1}H spectra of 11,23-Bis-((4-(((3'-hydroxy-3-oxo-3H-spiro[isobenzofuran-1,9'-xanthen]-6'-yl)oxy)methyl)-1H-1,2,3-triazol-1-yl)methyl)-25,27-dihydroxy-26,28-dioctyloxycalix[4]arene (**5**) (up) and it's mixture with Rh6G(bottom) (a); 2D NMR ^{1}H-^{1}H NOESY spectra of 5- Rh6G (b), C(5) = C Rh6G = 0.001 mM, DMSO-d6, 25 °C.

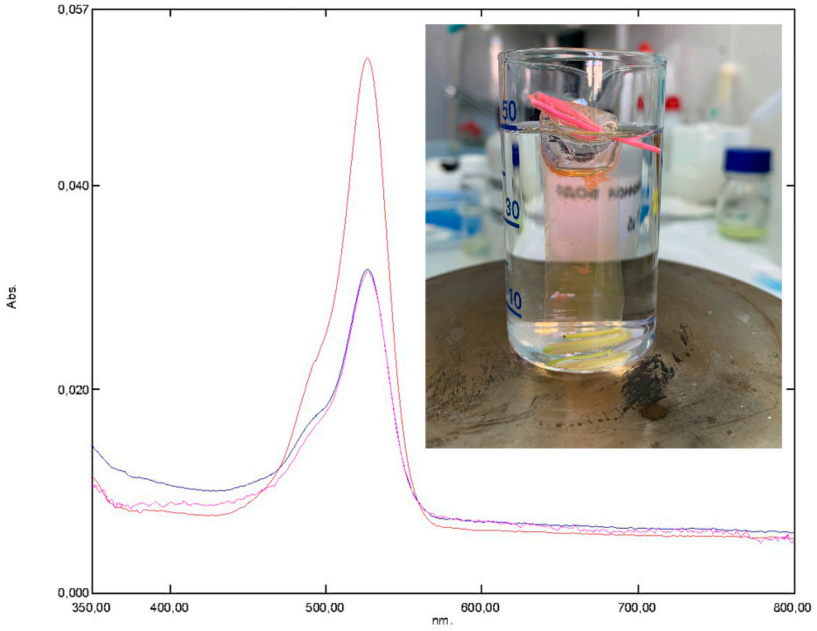

Figure S5. UV-vis spectra of aqueous solution of Rh6G before sorption (red line) and after 1 hour (black line) of 24 hours (cyan line) of sorption by SLN's and photography of the sorption experiment setup (insert). C(SLN's) = 1 mg/ml, V = 3 ml; C(Rh6G) = 0.007 mM, V = 50 ml

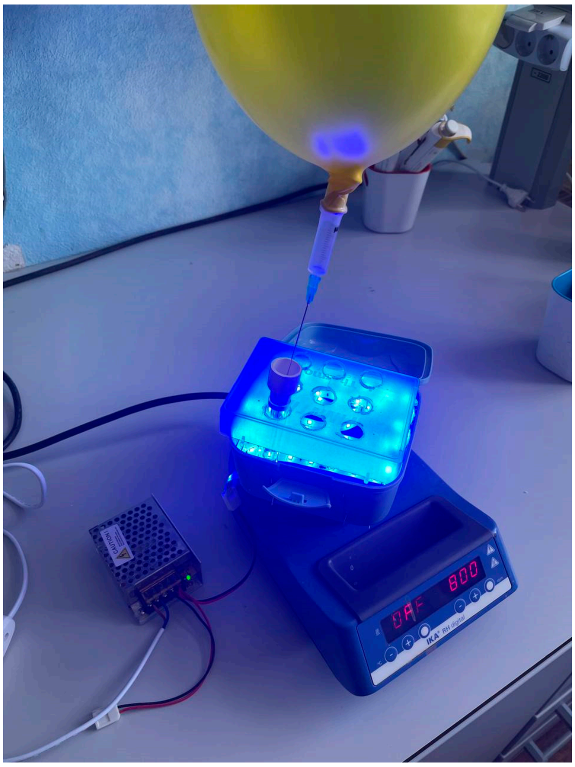

Figure S6. Photoredox blue-LED reactor setup.

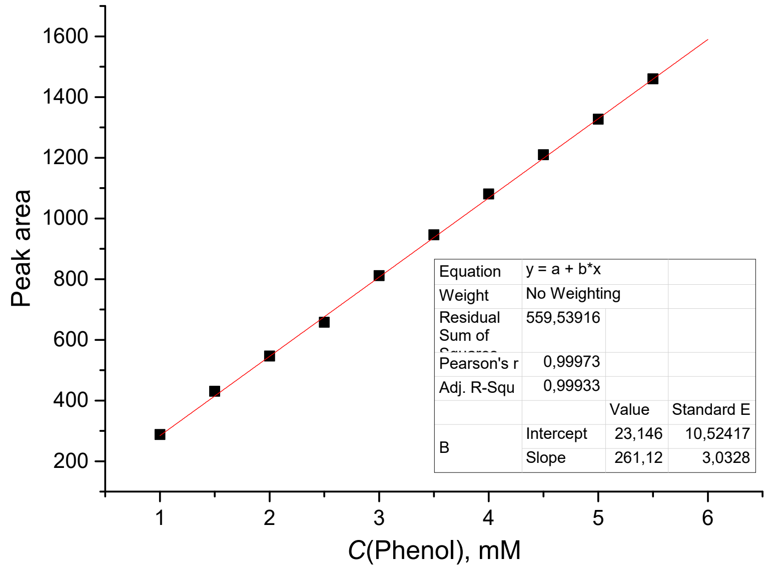

Figure S7. HPLC –UV calibration curve.

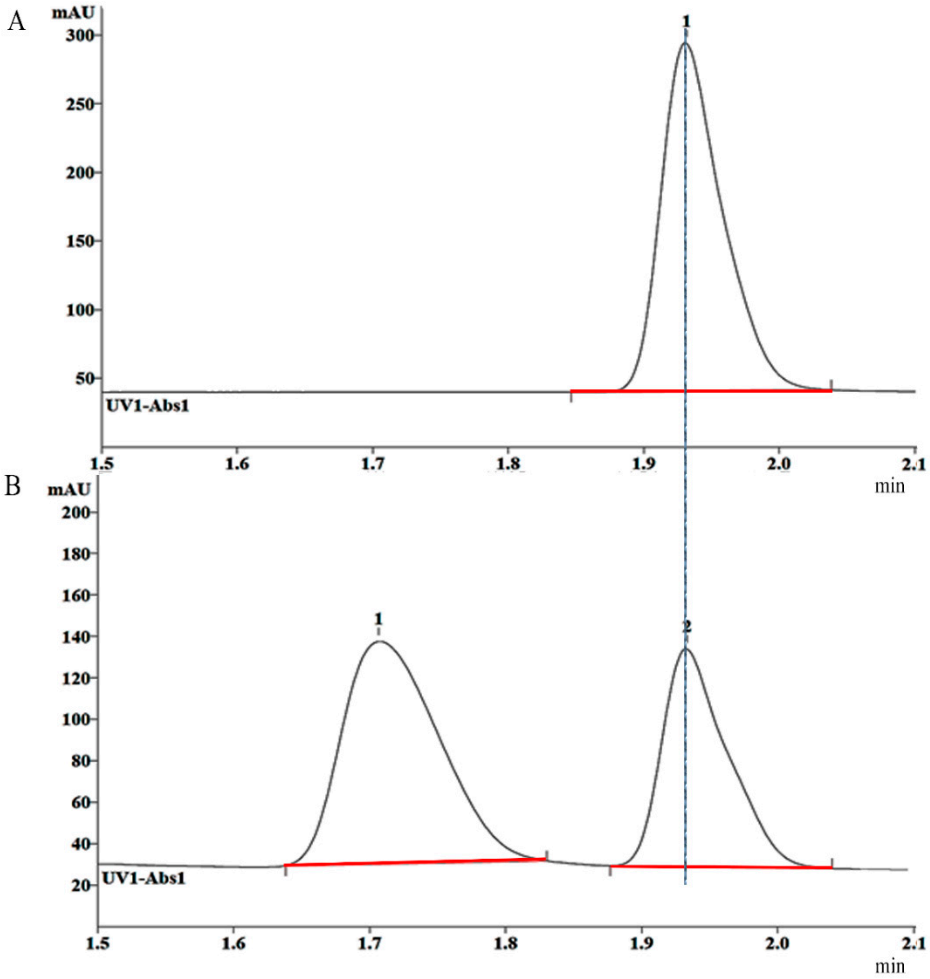

Figure S8. Chromatograms of pure phenol (A) and reaction mixture of phenol with phenylboronic acid (B) (Macherey-Nagel EC 250 / 4.6 NUCLEODUR C18 column using 1.5 ml/min CH_3CN-H_2O (80:20) eluent).

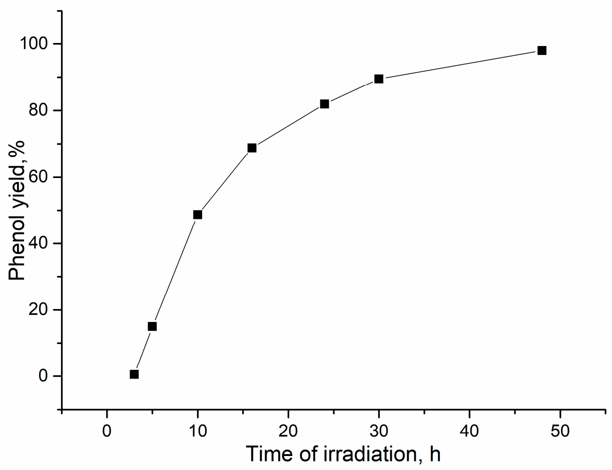

Figure S9. HPLS –UV yield of phenol from photoredox hydroxylation of phenylboronic acid using SLN's+Rh6G catalytic system *vs* time of irradiation, 0.1 mmol $PhB(OH)_2$, 0.2 mmol NEt_3, 0,7 µmol SLN's + 0,035 µmol Rh6G, blue LED7.45 W.
